# Deep sequencing reveals persistence of cell-associated mumps vaccine virus in chronic encephalitis

**DOI:** 10.1007/s00401-016-1629-y

**Published:** 2016-10-21

**Authors:** Sofia Morfopoulou, Edward T. Mee, Sarah M. Connaughton, Julianne R. Brown, Kimberly Gilmour, WK ‘Kling’ Chong, W. Paul Duprex, Deborah Ferguson, Mike Hubank, Ciaran Hutchinson, Marios Kaliakatsos, Stephen McQuaid, Simon Paine, Vincent Plagnol, Christopher Ruis, Alex Virasami, Hong Zhan, Thomas S. Jacques, Silke Schepelmann, Waseem Qasim, Judith Breuer

**Affiliations:** 1 Division of Infection and Immunity, University College London, Cruciform Building, Gower Street, London, WC1E 6BT UK; 2 Virology Division, National Institute for Biological Standards and Control, Potters Bar, EN6 3QG UK; 3 Microbiology, Virology and Infection Control, Great Ormond Street Hospital for Children, NHS Foundation Trust, London, WC1N 3JH UK; 4 Department of Immunology, Great Ormond Street Hospital, NHS Foundation Trust, London, WC1N 3JH UK; 5 Department of Radiology, Great Ormond Street Hospital for Children, NHS Foundation Trust, London, WC1N 3JH UK; 6 Department of Microbiology, Boston University School of Medicine, Boston, MA 02118 USA; 7 Genetics and Genomics Medicine UCL Great Ormond Street Institute of Child Health, London, WC1N 1EH UK; 8 Department of Histopathology, Great Ormond Street Hospital for Children, NHS Foundation Trust, London, WC1N 3JH UK; 9 Neurosciences Department, Great Ormond Street Hospital, NHS Foundation Trust, London, WC1N 3JH UK; 10 Molecular Pathology Programme, Centre for Cancer Research and Cell Biology, Queen’s University, Belfast, UK; 11 Tissue Pathology, Belfast Health and Social Care Trust, Belfast City Hospital, Lisburn Road, Belfast, UK; 12 Nottingham University Hospitals NHS Trust, Queen’s Medical Centre, Nottingham, NG7 2UH UK; 13 UCL Genetics Institute, University College London, London, WC1E 6BT UK; 14 Molecular and Cellular Immunology Section, UCL Great Ormond Street Institute of Child Health, London, WC1N 1EH UK; 15 Developmental Biology and Cancer Programme, UCL Great Ormond Street Institute of Child Health, London, WC1N 1EH UK; 16 Great Ormond Street Hospital for Children, NHS Foundation Trust, Molecular and Cellular Immunology, London, WC1N 3JH UK; 17 UCL Great Ormond Street Institute of Child Health, London, WC1N 1EH UK

## Abstract

Routine childhood vaccination against measles, mumps and rubella has virtually abolished virus-related morbidity and mortality. Notwithstanding this, we describe here devastating neurological complications associated with the detection of live-attenuated mumps virus Jeryl Lynn (MuV^JL5^) in the brain of a child who had undergone successful allogeneic transplantation for severe combined immunodeficiency (SCID). This is the first confirmed report of MuV^JL5^ associated with chronic encephalitis and highlights the need to exclude immunodeficient individuals from immunisation with live-attenuated vaccines. The diagnosis was only possible by deep sequencing of the brain biopsy. Sequence comparison of the vaccine batch to the MuV^JL5^ isolated from brain identified biased hypermutation, particularly in the matrix gene, similar to those found in measles from cases of SSPE. The findings provide unique insights into the pathogenesis of paramyxovirus brain infections.

**Electronic supplementary material:**

The online version of this article (doi:10.1007/s00401-016-1629-y) contains supplementary material, which is available to authorized users.

## Introduction

Chronic encephalitis with progressive loss of motor and cognitive function and high levels of intrathecal antibodies has been associated with persistent measles and rubella infections of the brain, most commonly in the context of subacute sclerosing pan-encephalitis (SSPE). We describe here a case of chronic panencephalitis in a child who had undergone successful allogeneic haematopoietic stem cell transplantation (allo-SCT) for severe combined immunodeficiency (SCID) in whom neither measles nor other pathogens could be detected. Using deep sequencing of fresh brain biopsy material, we identified the Jeryl Lynn 5 mumps virus (MuV^JL5^), a component of the measles, mumps, rubella (MMR) vaccine that had been administered to the child before the diagnosis of SCID. Similar to findings in measles viruses recovered from cases of SSPE, the mumps virus genome from the brain showed evidence of biased hypermutation, particularly in the matrix (M) gene. Comparison with sequence data from the original vaccine batch used to immunise this child identified the expansion of variants present at low frequency in the vaccine as well as de novo fixed missense substitutions. This case represents the first conclusive demonstration of chronic panencephalitis due to mumps virus.

## Case report

**Fig. 1:**
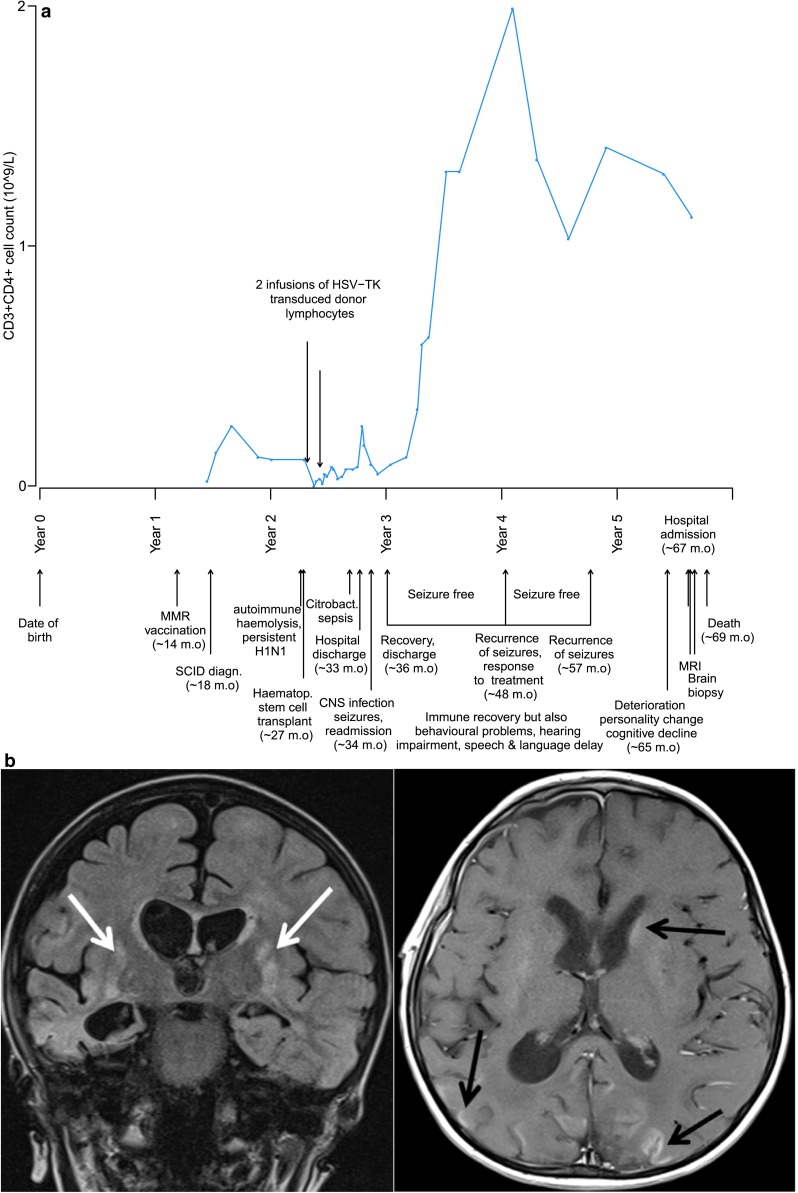
**a** Patient timeline presenting important clinical events (m.o. = months old) and immune recovery of CD3+ CD4+ T cells over this period. **b** MRI brain scan showing coronal T2-weighted FLAIR (FLuid Attenuated Inversion Recovery) and axial post-contrast T1-weighted images with bilateral basal ganglia lesions (*white arrows*) and enhancing cortical and deep grey matter lesions (*black arrows*). The pattern was typical of those described in subacute panencephalitis

An 18-month-old male infant of consanguineous parents was diagnosed with SCID due to recombinase activating gene 1 (RAG1) deficiency (Fig. 1a) 4 months after MMR vaccination. The infant received a CD34-selected haploidentical allo-SCT. Full donor chimerism was rapidly achieved, with recovery of a diverse T cell repertoire (Fig. 1a) and excellent thymopoiesis. Post-transplant autoimmune cytopenias necessitated rituximab therapy, following which immunoglobulin replacement therapy was administered. Six months post-allo-SCT the child developed a febrile illness with rash, diarrhoea, lethargy and seizures, with evidence of encephalitis on magnetic resonance imaging (MRI) with raised CSF protein (1.35 g/L) and 11 lymphocytes. Despite extensive screening for neurotropic viruses and bacteria (Table S1), no pathogen was detected. He was treated with antimicrobials, antivirals and steroids, making a good recovery, and was discharged on anticonvulsant therapy. Over the next few months, the child was noted to have behavioural problems, hearing impairment and speech and language delay. One year after discharge, the seizures recurred with only partial response to antiepileptic treatment, but he remained stable for another 9 months. Repeat MRI scan 2 years after initial encephalitic illness showed no new lesions. Over the next few months, the child’s neurological condition deteriorated, with increasing seizures together with episodes of lethargy, disorientation, agitation, ataxic gait, visual loss and eventual hospitalisation.

Repeat MRI of the brain at 40 months post-allo-SCT revealed abnormalities of grey and white matter, involving both cerebral hemispheres with multiple foci of contrast enhancement, including the basal ganglia, temporal and parieto-occipital cortices (Fig. 1b). In the absence of a firm diagnosis, a brain biopsy was taken.

Broad-spectrum antibiotics, aciclovir, ganciclovir and antifungal therapy were administered, as well as intravenous immunoglobulin (IVIG) and high-dose methyl prednisolone. Increasing seizures, left-sided weakness, cortical blindness and progressive global neurological deterioration over several weeks ended with the patient’s death 7 weeks following his last hospital admission.

## Materials and methods

### PCR assays

The viruses listed in Table S1 were tested for using real-time PCR and found to be negative using specific primers and TaqMan probes on an ABI 7500 thermocycler.

All PCR assays were performed in-house at GOSH (Great Ormond Street Hospital for Children, London) as part of the routine diagnostic service, unless indicated otherwise, in which case they were sent to a different laboratory for testing.

The CSF and urine samples collected during the patient’s last hospitalisation, as well as RNA re-extracted from the brain biopsy, were sent to the Public Health England Virus Reference Laboratory (Colindale) for mumps and mumps vaccine-specific RT-PCR (targeting the SH and HN gene) as well as measles and rubella-specific RT-PCR.

### Sequencing

#### Library preparation—brain biopsy

Total RNA was purified from the frozen brain biopsy and polyA RNA-separated for sequencing library preparation. Samples were sequenced on the Illumina NextSeq500 instrument (Illumina, San Diego, US) using an 81 bp paired-end run. Libraries to be multiplexed in the same run were pooled in equimolar quantities, calculated from qPCR and/or Bioanalyser fragment analysis.

Samples were processed using Illumina’s TruSeq Stranded mRNA LT sample preparation kit (p/n RS-122-2101) according to the manufacturer’s instructions. Deviations from the protocol were as follows: (1) 250 ng total RNA was used as starting material. (2) Fragmentation was carried out for 10 min instead of 8 min. (3) 14 Cycles of PCR were used.

Briefly, mRNA was isolated from total RNA using oligo dT beads to pull down poly-adenylated transcripts. The purified mRNA was fragmented using chemical fragmentation (heat and divalent metal cation) and primed with random hexamers. Strand-specific first-strand cDNA was generated using SuperScript II Reverse Transcriptase (Life Technologies) and actinomycin D. This allows RNA-dependent synthesis while preventing spurious DNA-dependent synthesis. The second cDNA strand was marked by performing synthesis incorporating dUTP.

The cDNA is then “A-tailed” at the 3′ end to prevent self-ligation during the addition of the full-length TruSeq Adaptors (adaptors have a complementary “T” overhang). The adaptors contain sequences that allow the libraries to be amplified by PCR, bind to the flow cell and be uniquely identified by way of a 6 bp index sequence. Finally, a PCR is carried out to amplify only those cDNA fragments that have adaptors bound to both ends.

#### Library preparation—vaccine

An archived vial from the batch of MMR vaccine used to immunise the child was deep sequenced in a 2 × 251 paired-end sequencing on an Illumina MiSeq as part of a 16-sample pool, generating 1,570,733 read pairs, at the National Institute for Biological Standards and Control (NIBSC).

Lyophilised vaccine was resuspended in 500 µl sterile water for injection and viral RNA extracted using the QIAamp Viral RNA Mini Spin Kit (Qiagen) according to the method of the manufacturer, with the omission of carrier RNA. DNA libraries were prepared by random reverse-transcriptase polymerase chain reaction as described previously [18], followed by fragmentation, adaptor addition and indexing using the Nextera XT library preparation kit (Illumina).

### Immunohistochemistry

Formalin-fixed paraffin-embedded sections of brain biopsy were examined by conventional histology. Immunohistochemistry was undertaken using two different antibodies in two independent laboratories.

Immunohistochemistry was undertaken using mumps nucleoprotein (N) antibody (7B10: sc-57921 Santa Cruz) at 1 in 25 using a Leica Bond Max automated staining system with pretreatment HIER (30) ER2. Additional immunohistochemistry was independently undertaken using a different antibody. The monoclonal mumps antibody used [16] recognises the N protein of MuV (N93-51/01) and was used on an automated Leica BondMax immunostainer at a dilution of 1/4000 following HIER2 pre-treatment for 20 min. Detection sites were detected with a polymer-based detection system (Bond, Newcastle upon Tyne, UK, Cat. No. DS9800). Detection of mumps N in the CNS by immunohistochemistry is shown in Figure S2.

### Bioinformatics analysis

For the analysis of the brain biopsy sequencing data, we implemented the following steps. We first removed duplicate sequences that can arise from PCR amplification with an in-house script that collapses pairs of reads based on sequence identity using 90 % of the sequence as signature (20 % removed as duplicates). Half of the reads overlapped with their “mates” within pairs and we therefore merged the overlapping reads using PEAR [32], taking into account both sequence match and quality scores. We performed quality control using PrinSeq [25], trimming low-quality ends and removing reads that had average quality less than 15. We subsequently removed human sequences, using a quick aligner (Novoalign version V2.07.13—human reference genome GRCh37) as well as BLASTn [2]. We performed de novo assembly of high-quality contigs using Velvet [31] (kmer = 81). Finally, we annotated the contigs and the unassembled reads against a custom protein reference database using BLASTx. Our custom protein reference database consists of viral, bacterial, human and mouse RefSeq proteins. More specifically, all known viruses in the RefSeq collection are used ftp://ftp.ncbi.nlm.nih.gov/refseq/release/viral/viral.1.protein.faa.gz, as well as all the bacteria of the human microbiome, according to ftp://ftp.ncbi.nih.gov/genomes/HUMAN_MICROBIOM/Bacteria/all.faa.tar.gz. The BLASTx results were the input of metaMix [21].

For the analysis of the vaccine sequencing, we first removed 15 % of the reads as duplicates and merged overlapping reads. We trimmed the reads based on base quality (*q* = 20) using Trim Galore! (http://www.bioinformatics.babraham.ac.uk/projects/trim_galore/). We then selected for Jeryl Lynn vaccine strain reads using BLASTn [2] and a Jeryl Lynn nucleotide reference database.

We performed de novo assembly with SPADeS [4] followed by QUAST [12] for both the vaccine and the brain sequencing data. In the latter case, we used the MuV reads as identified by metaMix [21]. Novoalign, Samtools [17] and VarScan2 [15] were used for consensus sequence generation and variant calling. We filtered variants based on quality, depth, frequency and strand bias (quality ≥30, at least 5 reads for the variant site, frequency ≥5 %, *p* value <0.01). The variants were annotated with SnpEff [7].

We compared the number of non-synonymous changes observed in each of the MuV genes to the number we would expect if the observed missense mutations were randomly distributed across the genome, correcting for the gene length. Significant deviation from the expected number of mutations was tested with the goodness-of-fit two-tailed exact binomial test. Analysis was conducted with the statistical language R (http://www.r-project.org).

We estimated a maximum likelihood phylogenetic tree using RAxML [27] and 64 full MuV genomes from GenBank (accessed on 20 June 2015).

## Results

**Fig. 2:**
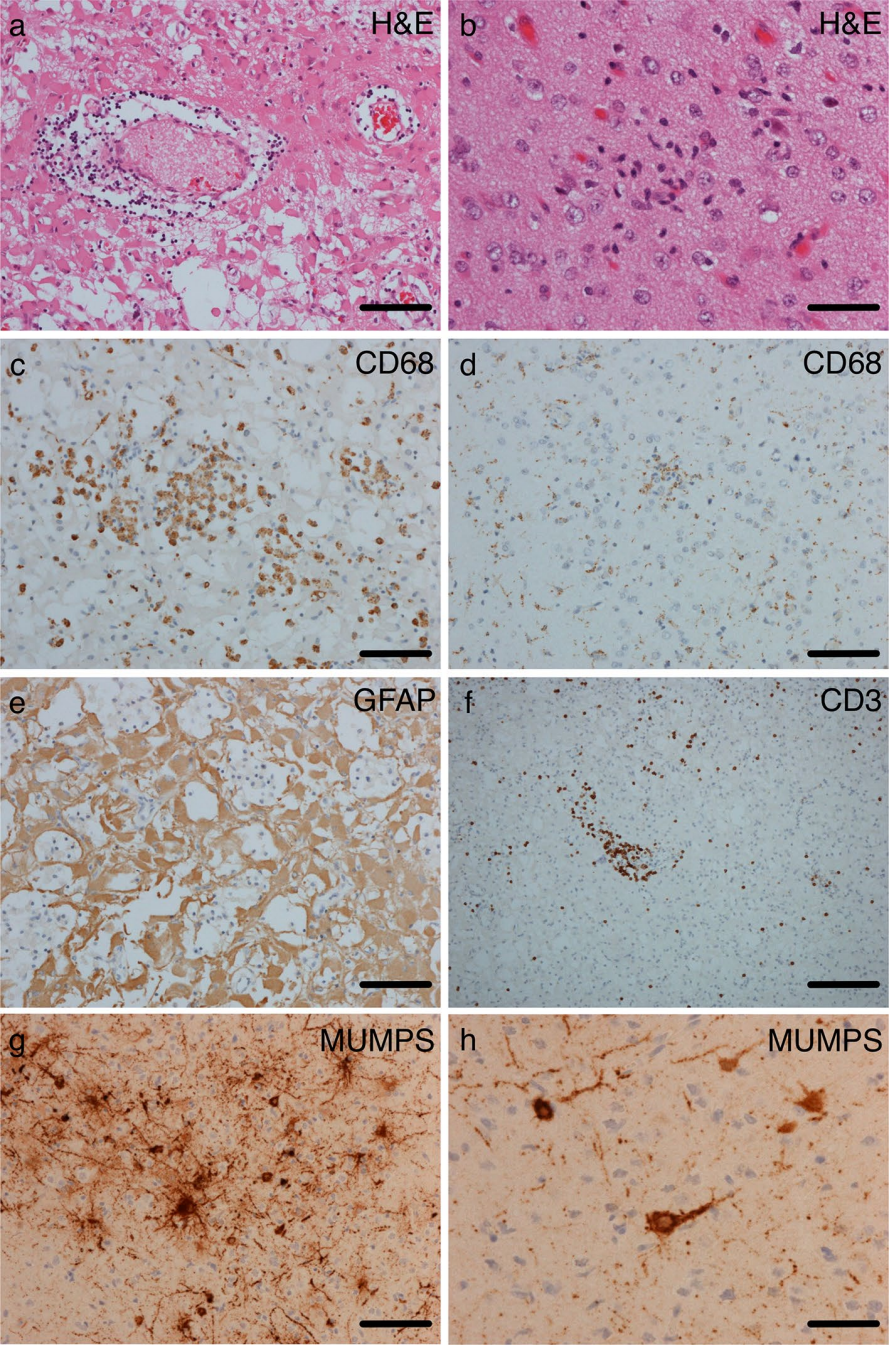
Much of the cortex showed significant tissue damage with neuronal loss. There was prominent reactive gliosis composed of plump astrocytosis with abundant eosinophilic cytoplasm and immunoreactivity for glial fibrillary acidic protein (GFAP) (**a, e**) with relative sparing of the superficial layers of the cortex. Some areas showed perivascular lymphocytosis and collections of macrophages (**a, c**). In others, with better neuronal preservation, there were foci of microglial nodules, neuronophagia, generalised microgliosis (demonstrated on CD68 staining **b, d**)) and patchy parenchymal and perivascular lymphocytes, the majority of which were positive for the T cell markers (**f**). Occasional foci of mineralisation were observed, but there was no vasculitis and no viral inclusions. The pathological changes extended into the underlying white matter (data not shown), which showed patchy loss of myelin staining on luxol fast blue. There was focal chronic inflammation in the leptomeninges. Mumps immunohistochemistry was positive in a neuronal pattern (**g, h**). We observed no specific staining for other pathogens HSV1 or 2, CMV, EBV, toxoplasma, JC virus, bacteria (Gram and Gram Twort), acid-fast bacteria (Ziehl-Neelson) or fungi (Grocott), and no evidence of intracellular or intranuclear viral inclusion bodies. **a**, **b** Haematoxylin and eosin (H&E), **c**, **d** CD68 immunohistochemistry, **e** GFAP immunohistochemistry, **f** CD3 immunohistochemistry. *Scale bars*-**b**, **h** 50 μm, **a**, **c**, **d**, **e**, **g** 100 μm, f 200 μm

CSF on last admission was acellular, but with oligoclonal bands, although total protein was low 0.12 g/L (normal 0.15–0.45 g/L. The oligoclonal bands were negative for HSV and VZV antibodies. Polyoma JC virus was detected by PCR in blood and urine on one occasion each (Table S2). CSF PCR was negative for 16S (bacterial) and 18S (fungal) rRNA and for a wide number of viral pathogens (Table S2), including JC virus, mumps, measles and rubella viruses. No pathogens were detected in stool, urine or blood (Table S2). CSF was negative for rubella and measles antibodies. In the absence of a firm diagnosis, a brain biopsy showed neuronal loss, astrocytic gliosis, reactive astrocytes, microglia and chronic inflammatory cells, but no viral inclusions or granulomata (Fig. 2a–e legend for detailed description). Infiltrating T lymphocytes were identified by CD3 staining (Fig. 2f).

RNA-Seq of the brain biopsy [5, 20] resulted in 110 million 81 base pair (bp) paired-end reads. Approximately, one million reads were identified as non-human and were subsequently used for potential pathogen identification by the metaMix method [21]. Mumps virus was the only potential pathogen detected, with 77,624 assigned reads. The remaining reads were either unclassified or mapped to human and bacteria that are either environmental, part of human flora, kit contaminants [23] or of unknown significance (Table S3).

No reads mapped to measles, rubella or polyoma JC viruses. Using de novo assembly, the full-length viral sequence was recovered (99.94 % genome coverage, median coverage depth: 290) (Figure S1). Maximum likelihood phylogenetic analysis showed that the consensus viral sequence clustered closely with the MuV^JL5^ vaccine strains (Figure S2) and shared 99.6 % identity with the publicly available sequence (GenBank: FJ211585) of the mumps component of the MMR preparation administered to the child. The sequence identified in this study is named MuV^JL5^-London (GenBank: KX223397). PCR of RNA extracted from the brain biopsy confirmed the presence of MuV^JL5^ vaccine strain (Table S2), but was negative for all other viruses, including polyoma JC (Table S2). Immunohistochemistry showed the presence of MuV nucleocapsid (N) protein with a neuronal pattern of staining (Fig. 2g, h). Control cortex and white matter were negative. We observed no specific staining for other pathogens (Fig. 2g,h for details) and no evidence of viral intracytoplasmic or intranuclear inclusion bodies. Retrospective testing showed weakly positive mumps antibody in the CSF.

To investigate this finding further, three million 251 bp paired-end reads were generated from the MMR vaccine batch that was used to immunise the child, of which 82,000 reads mapped to the MuV^JL5^ vaccine strain (median coverage: 706, Figure S1). There were no fixed differences identified compared with the reference FJ211585. Fifteen positions in the vaccine sequence were found to be polymorphic (variant allele frequency (VAF) 5 % or greater, Table S4).

**Fig. 3:**
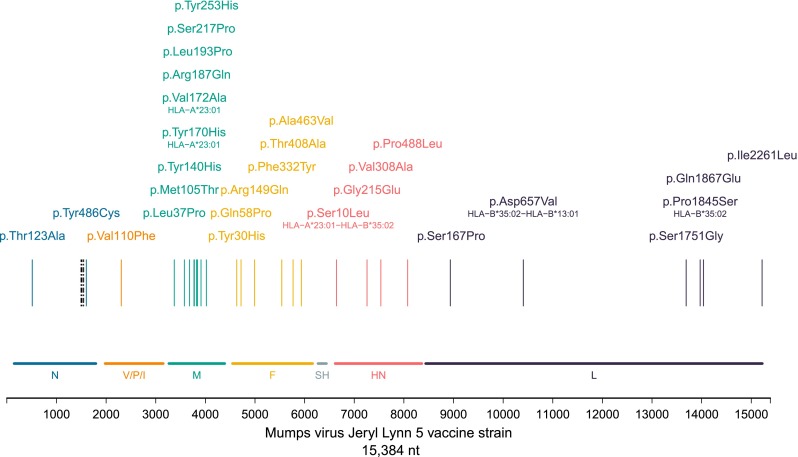
Fixed (defined to have frequency greater than 75 %) missense substitutions in the MuV^JL5^-London brain mumps virus genome. The amino acid changes are plotted along the genome, colour-coded for each viral protein. The *black dashed lines* are the missense changes pre-existing as minor variants in the genome of the vaccine virus. The predicted CTL epitopes are also indicated

Of 55 fixed nucleotide differences between MuV^JL5^-London and the vaccine, 12 had been present as a minority variant (10–24 %) in the vaccine (Figure S3, Table S4, VAF 75 % or greater). Of the remaining 43 new mutations, 28 coded for missense amino acid substitutions (Fig. 3, Table S5), with nine (32 %) located in the M protein, more than expected by chance (two-tailed exact binomial test *p* = 2.8e−04, Table S6). Other than position 140 in the M protein, the missense mutations were unique to MuV^JL5^-London (Figure S6). The Tyr140His substitution in MuV^JL5^-London is found in all wild-type strains as well as the more neurovirulent MuV^Ur^ Urabe and the MuV^JL2^ Jeryl Lynn 2 vaccine strain, which is a minor component of some vaccines [1] (Figure S6).

## Discussion

Prior to the introduction of routine MMR vaccination, mumps virus was the commonest cause of meningoencephalitis in children and was associated with serious sequelae including deafness and orchitis [11, 22]. No meningoencephalitis has been confirmed following the Jeryl lynn mumps vaccine, which this child received, and other adverse effects are rare [19] and usually self-limiting [14]. Mumps and rubella vaccine virus transcripts were detected in the brain of a child with IFNAR2 deficiency who developed fatal encephalitis following MMR vaccination [9], but brain immunohistochemistry was negative in this case (JB personal communication).

The presentation of this case, shares features with two previously published cases of MuV-related progressive encephalitis [13, 28]. Moreover, like measles SSPE and measles inclusion body encephalitis (MIBE) [29], which occurs in immunocompromised children within months of measles infection or vaccination, and unlike acute MuV encephalitis, no virus was detected in the CSF. However, other hallmarks of SSPE and MIBE such as high virus-specific antibody levels in CSF [29], were absent, although the child had deficient B cell function.

Importantly, however, in comparison with MuV^JL5^ from the vaccine batch used to immunise the child, MuV^JL5^-London showed evidence of T to C biased hypermutation, particularly in the M (matrix) gene suggestive of adenosine deaminase (ADAR)-driven mutation [10] (Table S4). The same finding in MV recovered from SSPE and MIBE [6, 29, 30] has been shown to reduce M protein expression [29] and is mooted to promote cell to cell spread and reduced viral assembly and shedding into the CSF. Biased hypermutation and lack of protein expression may also reflect the dispensability of M protein for neurovirulence [3].

MuV^JL5^-London was characterised by the expansion of variants present at low frequency in the original vaccine, two of which have previously been described [8], as well as fixed de novo mutations in the M, N, P, L and F genes, a finding which again references those described for MV in SSPE. While most amino acid substitutions occurred in the M gene, in vitro data using the rat model implicates the F protein [3, 16] as key for mumps virus neurovirulence in acute encephalitis [16, 24] as well as for measles neurovirulence in SSPE [3]. The absence of within-host sequence diversity in the M and F ORFs (Figure S5, Table S7) further suggests functional constraints on these genes and supports directional selection acting on one or more of the amino acid changes in these proteins favouring spread and replication of MuV^JL5^ in the brain.

In this child, clinical deterioration occurred following good T cell reconstitution in the context of normal T cell receptor repertoire and good levels of thymopoiesis. One possibility is that the presence of mumps tolerised and dysregulated T cell responses, thus permitting long-term viral persistence and the accumulation of pathogenic mutations. We made use of data on the donor’s HLA genotype to predict specific CTL cell epitopes (IEDB epitope prediction tool, percentile rank cutoff ≤1 %) in the MuV^JL5^ mumps vaccine virus. Five missense mutations were located in the predicted CTL epitopes for the donor’s HLA (Fig. 3, Table S8). Of these, four had predicted T cell binding affinities at a level likely to correlate with T cell recognition [26] and reduced binding affinity in the mutated peptide (Table S8). This raises the possibility that partial escape of virus from immune surveillance through specific mutational changes may have occurred, especially as the same was not observed for peptides recognised by randomly selected HLA alleles (Supplementary Appendix, Table S8).

In summary, we report a case of progressive chronic encephalitis in a patient with SCID, in which the MuV^JL5^ vaccine strain was detected in brain biopsy by deep sequencing. This case emphasises the generally poor rates of pathogen detection in encephalitides, making a strong case for deep sequencing of brain tissue where other methods have failed. The similar pattern of viral mutations in this case and those of measles SSPE suggest a common pathogenic process and further work is currently underway to determine the contribution of fixed mutations to the chronic encephalitic phenotype observed in the patient. It is important to note that MMR continues to be a highly effective and safe vaccine in the vast majority of individuals. However, this case highlights the importance of developing strategies such as newborn screening to exclude the very small proportion of infants at extremely high risk of complications from live-attenuated vaccines.

## Electronic supplementary material

Below is the link to the electronic supplementary material.

Supplementary material 1 (DOCX 3324 kb)
