# Supplementary material for: Deep sequencing reveals persistence of cell-associated mumps vaccine virus in chronic encephalitis
Source: Acta Neuropathol. 2016 Oct 21;133(1):139–47. doi: 10.1007/s00401-016-1629-y (PMC5209397; doi:10.1007/s00401-016-1629-y)
Supplement: Supplementary file 1 — Supplementary material 1 (DOCX 3324 kb) [file 401_2016_1629_MOESM1_ESM.docx]

# Deep sequencing reveals persistence of cell-associated mumps vaccine virus in chronic encephalitis

Sofia Morfopoulou, Edward T Mee, Sarah M Connaughton, Julianne R Brown, Kimberly Gilmour, WK ‘Kling’ Chong, W Paul Duprex, Deborah Ferguson, Mike Hubank, Ciaran Hutchinson, Marios Kaliakatsos, Stephen McQuaid, Simon Paine, Vincent Plagnol, Chris Ruis, Alex Virasami, Hong Zhan, Thomas S Jacques, Silke Schepelmann, Waseem Qasim, Judith Breuer

Contents

[Deep sequencing reveals persistence of cell-associated mumps vaccine virus in chronic encephalitis 1](#_Toc463265450)

[Single nucleotide polymorphisms detection 2](#_Toc463265451)

[Epitope prediction 2](#_Toc463265452)

[Supplementary Tables 3](#_Toc463265453)

[Supplementary Figures 10](#_Toc463265454)

## Single nucleotide polymorphisms detection

Fifteen positions in the vaccine sequence were found to be polymorphic (MAF 5% or greater, **Table S6b and S6c**). These were all located in the N gene which codes for the nucleocapsid protein or in the untranslated region between the N and the V/P/I genes. The majority of the changes were T to C substitutions. Five of the changes coded for missense amino acid substitutions, five were synonymous while the remaining five changes were in non-coding regions. Twelve polymorphisms were present at frequencies of 10-24%.

Eighty one differences between the brain MuV data and the vaccine consensus genome were observed (MAF 5% or greater, **Figure S3, Table S6a**) with an excess of T to C changes (51/81). Fifty five of these differences had become fixed in the brain MuV consensus sequence (**Table S3,** MAF 75% or greater) while twenty six were minority variants (**Table S5)** and clustered at the 3’ends of the N and large (L) genes (**Figure S3**). Twelve changes were identical to the pre-existing minority variants identified at 10-24% frequency in or near to the N gene, now fixed in the brain virus. These were all T to C changes, four of them predicted to result in non-synonymous changes, three in synonymous and five was located in a non-coding region (**Table S6b, Figure S3**).

## Epitope prediction

We made use of data on the donor’s HLA genotype to predict specific CTL cell epitopes. We used the IEDB epitope prediction tool and the recommended percentile rank cutoff <= 1%. Five of the fixed missense changes in the M, HN, L proteins (2, 1 and 2 changes respectively) occurred within predicted CTL epitopes while none were found in N, P and F proteins. 4 out of the 5 epitopes are considered as peptide binders according to IEDB standards (IC50 score less than 500nM). A low IC50 value signifies a high binding affinity. In 4/5 cases the substitution present in the MuV^JL5^-London is predicted to reduce CTL binding affinity (Table S8). We repeated the analysis using four randomly selected HLA alleles. 4/6 of the predicted epitopes would not be regarded as peptide binders (Table S8). The mutations increased the binding affinity in half of the epitopes, while decreased it in the other half, without modifying their peptide binder status (Table S8). No loss of predicted T cell binding affinity seen in mutated peptides from two predicted epitopes based on four randomly selected HLA A and B alleles.

## Supplementary Tables

**Supplementary Table 1**: Summary of real-time PCR assays undertaken on the CSF at the first signs of CNS infection (patient was approximately 34 months old). All assays listed were negative.

**Supplementary Table 2**: Summary of real-time PCR assays undertaken on the brain biopsy, CSF, blood and urine samples (patient was approximately 67 months old). All assays listed were negative except for the mumps virus PCR assay for the brain biopsy which was positive as well as the JC virus PCR assay for one blood and one urine sample.

*Micropathology Laboratory Ltd., **Public Health England (PHE)

**Supplementary Table 3:** Summary of present species detected by metaMix. The only viral pathogen detected is MuV. Human sequences as well as environmental bacteria and kit contaminants were also detected. The posterior probability of the presence of the taxon in the sample is presented and the Bayes Factor to quantify the evidence in favor of the presence of each species to it being absent.

**Supplementary Table 4:** Summary of all 81 variants between the brain and the vaccine data consisting of 55 fixed and 26 minority variants.

**Supplementary Table 5:** 55 fixed mutations (frequency > 75%) between brain and vaccine data. Of these 50 occur in coding regions of the mumps virus genome, reported here per protein. The changes in the N gene present in the vaccine sequencing data as minority variants have a grey text background.

**Supplementary Table 6:** Goodness-of-Fit exact binomial test two-tailed p-value for significant deviation from the expected number of fixed missense changes per protein.

**Supplementary Table 7:** The 26 minority variants in the brain data consist of 17 changes in coding areas and 9 in non-coding. The table summarizes the former, reported per protein.

**Supplementary Table 8:** Epitope binding affinity scores (IC50) for the donor and random alleles. The asterisk (*) indicates peptide binders recognized by T cells (IC50<500nM).

**Supplementary Table 1**

| CSF |
| --- |
| Adenovirus |
| BK virus |
| JC virus |
| CMV |
| EBV |
| Enterovirus |
| Parechovirus |
| HHV6 |
| HSV |
| Toxoplasma |
| 16S (pan-bacterial) |
| 18S (pan-fungal) |
| Mycobacteria |

**Supplementary Table 2**

| Brain biopsy | CSF | Blood | Urine |
| --- | --- | --- | --- |
| Adenovirus | Adenovirus | Adenovirus |  |
| BK virus | BK virus | BK virus | BK virus |
| CMV | CMV | CMV | CMV |
| HHV6* | HHV6* |  |  |
| HSV 1 and 2 | HSV 1 and 2 |  |  |
| Human astrovirus | Human astrovirus |  |  |
| JC virus | JC virus | JC virus | JC virus |
| Measles** | Measles** |  | Measles** |
| Mumps** | Mumps** |  | Mumps** |
| Rubella** | Rubella** |  |  |
| EBV |  | EBV |  |
| Toxoplasma* |  | Toxoplasma* |  |
| Norovirus |  |  |  |
| Rotavirus |  |  |  |
| Sapovirus |  |  |  |
|  | Astrovirus VA1/HMO-C |  |  |
|  | Coronavirus* |  |  |
|  | Enterovirus* |  |  |
|  | Mycoplasma* |  |  |
|  | Parechovirus* |  |  |
|  | Rhinovirus* |  |  |
|  | RSV* |  |  |
|  | VZV |  |  |

**Supplementary Table 3**

| taxonID | scientName | Read count | poster.prob | log10BF |
| --- | --- | --- | --- | --- |
| unknown | unknown | 348908 | 1 | NA |
| 9606 | *Homo sapiens* | 101050 | 1 | 110573 |
| 11161 | *Mumps virus* | 77624 | 1 | 335240.4 |
| 1747 | *Propionibacterium acnes* | 2653 | 1 | 3339.266 |
| 10090 | *Mus musculus* | 1190 | 1 | 727.4473 |
| 374840 | *Enterobacteria phage phiX174 sensu lato* | 423 | 1 | 846.6118 |
| 584 | *Proteus mirabilis* | 421 | 1 | 624.2779 |
| 548476 | *Corynebacterium aurimucosum ATCC 700975* | 350 | 1 | 442.3845 |
| 47229 | *Massilia timonae* | 316 | 1 | 497.8983 |
| 529507 | *Proteus mirabilis HI4320* | 263 | 1 | 292.9151 |
| 158877 | *Yokenella regensburgei* | 256 | 1 | 241.8602 |
| 29388 | *Staphylococcus capitis* | 249 | 1 | 342.6923 |
| 33032 | *Anaerococcus lactolyticus* | 234 | 1 | 372.4314 |
| 469 | *Acinetobacter* | 232 | 1 | 326.9754 |
| 102862 | *Proteus penneri* | 223 | 1 | 201.0023 |
| 28037 | *Streptococcus mitis* | 223 | 1 | 314.1208 |
| 1282 | *Staphylococcus epidermidis* | 222 | 1 | 371.8281 |
| 40214 | *Acinetobacter johnsonii* | 206 | 1 | 308.6753 |
| 69218 | *Enterobacter cancerogenus* | 202 | 1 | 215.8242 |
| 225324 | *Enhydrobacter aerosaccus* | 198 | 1 | 466.9199 |
| 816 | *Bacteroides* | 178 | 1 | 407.5874 |
| 618 | *Serratia odorifera* | 175 | 1 | 268.8794 |
| 38303 | *Corynebacterium pseudogenitalium* | 171 | 1 | 268.9854 |
| 1303 | *Streptococcus oralis* | 169 | 1 | 285.8656 |
| 28090 | *Acinetobacter lwoffii* | 168 | 0.99 | 308.9395 |
| 1260 | *Finegoldia magna* | 164 | 1 | 355.4904 |
| 1270 | *Micrococcus luteus* | 161 | 1 | 371.4328 |
| 1833 | *Rhodococcus erythropolis* | 127 | 1 | 310.9948 |
| 38284 | *Corynebacterium accolens* | 126 | 0.93 | 84.55722 |
| 267747 | *Propionibacterium acnes KPA171202* | 118 | 1 | 221.4295 |
| 33029 | *Anaerococcus hydrogenalis* | 116 | 0.94 | 40.6264 |
| 1305 | *Streptococcus sanguinis* | 107 | 1 | 296.4448 |
| 13690 | *Sphingobium yanoikuyae* | 107 | 0.97 | 170.1415 |
| 39791 | *Corynebacterium glucuronolyticum* | 102 | 0.99 | 72.87183 |
| 250 | *Chryseobacterium gleum* | 97 | 1 | 278.5301 |
| 82347 | *Facklamia languida* | 97 | 1 | 309.0609 |
| 40215 | *Acinetobacter junii* | 93 | 0.94 | 78.6702 |
| 553199 | *Propionibacterium acnes SK137* | 80 | 0.96 | 123.512 |
| 186802 | *Clostridiales* | 75 | 0.98 | 45.65406 |
| 1656 | *Actinomyces viscosus* | 73 | 0.99 | 91.91713 |
| 28127 | *Prevotella buccalis* | 70 | 1 | 249.1014 |
| 1351 | *Enterococcus faecalis* | 69 | 1 | 234.7038 |
| 1155766 | *Enterococcus faecium Aus0004* | 60 | 1 | 225.3758 |
| 43675 | *Rothia mucilaginosa* | 56 | 0.99 | 187.2995 |

**Supplementary Table 4**

| 4a. Brain data against vaccine consensus | | | | |  |  |  |  | |
| --- | --- | --- | --- | --- | --- | --- | --- | --- | --- |
| Position | **Ref** | **Reads1** | **Var** | **Reads2** | **VarFreq** | **Effect** | **Gene** | **Codon change** | **Amino acid change** |
| 512 | A | 3 | G | 1313 | 99.7 | missense | N | c.367A>G | p.Thr123Ala |
| 1463 | G | 3363 | A | 378 | 10.1 | missense | N | c.1318G>A | p.Asp440Asn |
| 1496 | T | 2 | C | 1866 | 99.89 | missense | N | c.1351T>C | p.Phe451Leu |
| 1514 | T | 1 | C | 1354 | 99.93 | synonymous | N | c.1369T>C | p.Leu457Leu |
| 1515 | T | 0 | C | 1352 | 100 | missense | N | c.1370T>C | p.Leu457Ser |
| 1537 | T | 2 | C | 1171 | 99.74 | synonymous | N | c.1392T>C | p.Gly464Gly |
| 1547 | T | 2 | C | 1238 | 99.84 | missense | N | c.1402T>C | p.Phe468Leu |
| 1548 | T | 0 | C | 1372 | 99.93 | missense | N | c.1403T>C | p.Phe468Ser |
| 1590 | T | 3146 | C | 223 | 6.62 | missense | N | c.1445T>C | p.Phe482Ser |
| 1602 | A | 782 | G | 2641 | 77.15 | missense | N | c.1457A>G | p.Tyr486Cys |
| 1647 | A | 3849 | C | 238 | 5.82 | missense | N | c.1502A>C | p.Asn501Thr |
| 1731 | T | 2518 | C | 1975 | 43.96 | missense | N | c.1586T>C | p.Val529Ala |
| 1769 | T | 1 | C | 4173 | 99.98 | synonymous | N | c.1624T>C | p.Leu542Leu |
| 1801 | T | 2055 | C | 1442 | 41.22 | non-coding |  |  |  |
| 1815 | T | 0 | C | 3025 | 100 | non-coding |  |  |  |
| 1856 | T | 1 | C | 1113 | 99.91 | non-coding |  |  |  |
| 1860 | T | 1 | C | 913 | 99.89 | non-coding |  |  |  |
| 1873 | T | 1 | C | 280 | 99.64 | non-coding |  |  |  |
| 1882 | T | 1 | C | 105 | 99.06 | non-coding |  |  |  |
| 2306 | G | 0 | T | 1169 | 99.91 | missense | V/P/I | c.328G>T | p.Val110Phe |
| 3213 | T | 120 | C | 19 | 13.67 | non-coding |  |  |  |
| 3215 | T | 104 | C | 20 | 16.13 | non-coding |  |  |  |
| 3220 | T | 95 | C | 19 | 16.67 | non-coding |  |  |  |
| 3256 | T | 589 | C | 41 | 6.5 | non-coding |  |  |  |
| 3273 | T | 726 | C | 52 | 6.68 | missense | M | c.10T>C | p.Ser4Pro |
| 3373 | T | 51 | C | 917 | 94.73 | missense | M | c.110T>C | p.Leu37Pro |
| 3577 | T | 28 | C | 799 | 96.61 | missense | M | c.314T>C | p.Met105Thr |
| 3629 | T | 32 | C | 783 | 95.96 | synonymous | M | c.366T>C | p.Asp122Asp |
| 3681 | T | 34 | C | 842 | 96.12 | missense | M | c.418T>C | p.Tyr140His |
| 3771 | T | 18 | C | 479 | 96.38 | missense | M | c.508T>C | p.Tyr170His |
| 3778 | T | 22 | C | 450 | 95.34 | missense | M | c.515T>C | p.Val172Ala |
| 3786 | T | 16 | C | 415 | 96.29 | synonymous | M | c.523T>C | p.Leu175Leu |
| 3806 | T | 16 | C | 421 | 96.34 | synonymous | M | c.543T>C | p.Ser181Ser |
| 3823 | G | 0 | A | 475 | 100 | missense | M | c.560G>A | p.Arg187Gln |
| 3841 | T | 18 | C | 589 | 97.03 | missense | M | c.578T>C | p.Leu193Pro |
| 3911 | T | 26 | C | 594 | 95.65 | synonymous | M | c.648T>C | p.Asp216Asp |
| 3912 | T | 27 | C | 511 | 94.98 | missense | M | c.649T>C | p.Ser217Pro |
| 4020 | T | 28 | C | 590 | 95.47 | missense | M | c.757T>C | p.Tyr253His |
| 4575 | C | 1 | T | 382 | 99.74 | synonymous | F | c.30C>T | p.Gly10Gly |
| 4633 | T | 0 | C | 448 | 100 | missense | F | c.88T>C | p.Tyr30His |
| 4718 | A | 1 | C | 494 | 99.8 | missense | F | c.173A>C | p.Gln58Pro |
| 4991 | G | 1 | A | 410 | 99.76 | missense | F | c.446G>A | p.Arg149Gln |
| 5540 | T | 0 | A | 577 | 100 | missense | F | c.995T>A | p.Phe332Tyr |
| 5767 | A | 0 | G | 904 | 99.89 | missense | F | c.1222A>G | p.Thr408Ala |
| 5933 | C | 2 | T | 850 | 99.77 | missense | F | c.1388C>T | p.Ala463Val |
| 6642 | C | 0 | T | 234 | 100 | missense | HN | c.29C>T | p.Ser10Leu |
| 6898 | A | 0 | G | 190 | 100 | synonymous | HN | c.285A>G | p.Gly95Gly |
| 6944 | T | 228 | C | 28 | 10.94 | synonymous | HN | c.331T>C | p.Leu111Leu |
| 6945 | T | 193 | C | 30 | 13.45 | missense | HN | c.332T>C | p.Leu111Ser |
| 7228 | T | 1 | C | 285 | 99.65 | synonymous | HN | c.615T>C | p.His205His |
| 7257 | G | 0 | A | 260 | 100 | missense | HN | c.644G>A | p.Gly215Glu |
| 7372 | C | 0 | T | 292 | 100 | synonymous | HN | c.759C>T | p.Cys253Cys |
| 7536 | T | 2 | C | 281 | 98.6 | missense | HN | c.923T>C | p.Val308Ala |
| 7756 | C | 2 | T | 606 | 99.67 | synonymous | HN | c.1143C>T | p.Val381Val |
| 8076 | C | 26 | T | 423 | 94.21 | missense | HN | c.1463C>T | p.Pro488Leu |
| 8936 | T | 0 | C | 70 | 100 | missense | L | c.499T>C | p.Ser167Pro |
| 10387 | T | 0 | C | 91 | 100 | synonymous | L | c.1950T>C | p.Asn650Asn |
| 10405 | T | 0 | C | 62 | 100 | synonymous | L | c.1968T>C | p.Asp656Asp |
| 10407 | A | 4 | T | 50 | 92.59 | missense | L | c.1970A>T | p.Asp657Val |
| 10414 | T | 0 | C | 70 | 100 | synonymous | L | c.1977T>C | p.Phe659Phe |
| 11431 | C | 0 | T | 118 | 100 | synonymous | L | c.2994C>T | p.Ser998Ser |
| 12379 | C | 4 | A | 46 | 92 | synonymous | L | c.3942C>A | p.Ser1314Ser |
| 13688 | A | 10 | G | 77 | 88.51 | missense | L | c.5251A>G | p.Ser1751Gly |
| 13970 | C | 0 | T | 100 | 100 | missense | L | c.5533C>T | p.Pro1845Ser |
| 14036 | C | 0 | G | 102 | 99.03 | missense | L | c.5599C>G | p.Gln1867Glu |
| 14500 | T | 0 | C | 123 | 100 | synonymous | L | c.6063T>C | p.Phe2021Phe |
| 14616 | T | 121 | C | 8 | 6.2 | missense | L | c.6179T>C | p.Leu2060Pro |
| 14868 | T | 169 | C | 11 | 6.11 | missense | L | c.6431T>C | p.Leu2144Pro |
| 14985 | T | 199 | C | 23 | 10.36 | missense | L | c.6548T>C | p.Leu2183Pro |
| 15008 | T | 222 | C | 16 | 6.72 | missense | L | c.6571T>C | p.Tyr2191His |
| 15012 | T | 237 | C | 16 | 6.32 | missense | L | c.6575T>C | p.Leu2192Pro |
| 15023 | T | 213 | C | 24 | 10.13 | synonymous | L | c.6586T>C | p.Leu2196Leu |
| 15157 | A | 363 | G | 37 | 9.25 | synonymous | L | c.6720A>G | p.Glu2240Glu |
| 15163 | T | 363 | C | 28 | 7.16 | synonymous | L | c.6726T>C | p.Ser2242Ser |
| 15174 | A | 362 | G | 21 | 5.48 | missense | L | c.6737A>G | p.Asp2246Gly |
| 15190 | A | 294 | G | 85 | 22.43 | missense | L | c.6753A>G | p.Ile2251Met |
| 15218 | A | 0 | C | 341 | 99.71 | missense | L | c.6781A>C | p.Ile2261Leu |
| 15248 | T | 253 | C | 153 | 37.68 | non-coding |  |  |  |
| 15314 | T | 173 | C | 58 | 25.11 | non-coding |  |  |  |
| 15317 | T | 89 | C | 78 | 46.71 | non-coding |  |  |  |
| 15318 | A | 154 | G | 49 | 24.14 | non-coding |  |  |  |

| 4b. Variants prexisting in the vaccine data | | | | |  |  |  |  |  |
| --- | --- | --- | --- | --- | --- | --- | --- | --- | --- |
| Position | **Ref** | **Reads1** | **Var** | **Reads2** | **Freq** | **Effect** | **Gene** | **Codon_change** | **Amino_acid change** |
| 1496 | T | 982 | C | 242 | 19.77% | missense | N | c.1351T>C | p.Phe451Leu |
| 1514 | T | 913 | C | 212 | 18.84% | synonymous | N | c.1369T>C | p.Leu457Leu |
| 1515 | T | 900 | C | 216 | 19.35% | missense | N | c.1370T>C | p.Leu457Ser |
| 1537 | T | 783 | C | 192 | 19.63% | synonymous | N | c.1392T>C | p.Gly464Gly |
| 1547 | T | 782 | C | 209 | 21.09% | missense | N | c.1402T>C | p.Phe468Leu |
| 1548 | T | 750 | C | 238 | 24.09% | missense | N | c.1403T>C | p.Phe468Ser |
| 1769 | T | 503 | C | 95 | 15.89% | synonymous | N | c.1624T>C | p.Leu542Leu |
| 1815 | T | 413 | C | 91 | 18.06% |  |  |  |  |
| 1856 | T | 373 | C | 37 | 9.02% |  |  |  |  |
| 1860 | T | 368 | C | 41 | 10.02% |  |  |  |  |
| 1873 | T | 367 | C | 42 | 10.27% |  |  |  |  |
| 1882 | T | 325 | C | 50 | 13.33% |  |  |  |  |

| 4c.Variants present only in the vaccine data | | | | |  |  |  |  |  |
| --- | --- | --- | --- | --- | --- | --- | --- | --- | --- |
| Position | **Ref** | **Reads1** | **Var** | **Reads2** | **Freq** | **Effect** | **GENE** | **Codon_change** | **Amino_acid change** |
| 1627 | T | 780 | C | 50 | 6.02% | synonymous | N | c.1482T>C | p.Asn494Asn |
| 2416 | A | 785 | C | 85 | 9.77% | synonymous | V/P/I | c.438A>C | p.Ser146Ser |
| 10494 | C | 226 | T | 13 | 5.44% | missense | L | c.2057C>T | p.Ala686Val |

**Supplementary Table 5**

| N (dN/dS=6/3) | | | P | | | M (dN/dS=9/4) | | |
| --- | --- | --- | --- | --- | --- | --- | --- | --- |
| Codon change | **Amino acid change** | **%** | **Codon change** | **Amino acid change** | **%** | **Codon change** | **Amino acid change** | **%** |
| c.367A>G | p.Thr123Ala | 100% | c.328G>T | p.Val110Phe | 99.91 | c.110T>C | p.Leu37Pro | 94.73 |
| c.1351T>C | p.Phe451Leu | 99.7 |  |  |  | c.314T>C | p.Met105Thr | 96.61 |
| c.1369T>C | p.Leu457Leu | 99.89 |  |  |  | c.366T>C | p.Asp122Asp | 95.96 |
| c.1370T>C | p.Leu457Ser | 99.93 |  |  |  | c.418T>C | p.Tyr140His | 96.12 |
| c.1392T>C | p.Gly464Gly | 100 |  |  |  | c.508T>C | p.Tyr170His | 96.38 |
| c.1402T>C | p.Phe468Leu | 99.74 |  |  |  | c.515T>C | p.Val172Ala | 95.34 |
| c.1403T>C | p.Phe468Ser | 99.84 |  |  |  | c.523T>C | p.Leu175Leu | 96.29 |
| c.1457A>G | p.Tyr486Cys | 99.93 |  |  |  | c.543T>C | p.Ser181Ser | 96.34 |
| c.1624T>C | p.Leu542Leu | 77.15 |  |  |  | c.560G>A | p.Arg187Gln | 100 |
|  |  |  |  |  |  | c.578T>C | p.Leu193Pro | 97.03 |
|  |  |  |  |  |  | c.648T>C | p.Asp216Asp | 95.65 |
|  |  |  |  |  |  | c.649T>C | p.Ser217Pro | 94.98 |
|  |  |  |  |  |  | c.757T>C | p.Tyr253His | 95.47 |

| F (dN/dS=6/1) | | | HN (dN/dS=4:4) | | | L (dN/dS:6:6) | | |
| --- | --- | --- | --- | --- | --- | --- | --- | --- |
| Codon change | **Amino acid change** | **%** | **Codon change** | **Amino acid change** | **%** | **Codon change** | **Amino acid change** | **%** |
| c.30C>T | p.Gly10Gly | 99.74 | c.29C>T | p.Ser10Leu | 100 | c.499T>C | p.Ser167Pro | 100 |
| c.88T>C | p.Tyr30His | 100 | c.285A>G | p.Gly95Gly | 100 | c.1950T>C | p.Asn650Asn | 100 |
| c.173A>C | p.Gln58Pro | 99.8 | c.615T>C | p.His205His | 99.65 | c.1968T>C | p.Asp656Asp | 100 |
| c.446G>A | p.Arg149Gln | 99.76 | c.644G>A | p.Gly215Glu | 100 | c.1970A>T | p.Asp657Val | 92.59 |
| c.995T>A | p.Phe332Tyr | 100 | c.759C>T | p.Cys253Cys | 100 | c.1977T>C | p.Phe659Phe | 100 |
| c.1222A>G | p.Thr408Ala | 99.89 | c.923T>C | p.Val308Ala | 98.6 | c.2994C>T | p.Ser998Ser | 100 |
| c.1388C>T | p.Ala463Val | 99.77 | c.1143C>T | p.Val381Val | 99.67 | c.3942C>A | p.Ser1314Ser | 92 |
|  |  |  | c.1463C>T | p.Pro488Leu | 94.21 | c.5251A>G | p.Ser1751Gly | 88.51 |
|  |  |  |  |  |  | c.5533C>T | p.Pro1845Ser | 100 |
|  |  |  |  |  |  | c.5599C>G | p.Gln1867Glu | 99.03 |
|  |  |  |  |  |  | c.6063T>C | p.Phe2021Phe | 100 |
|  |  |  |  |  |  | c.6781A>C | p.Ile2261Leu | 99.71 |

**Supplementary Table 6**

| Gene | significant deviation from expected number of fixed missense changes (p-value) | T to C hypermutation rate |
| --- | --- | --- |
| N | 0.77 | 62% (8/13) |
| P | 0.73 | 0% (0/1) |
| M | 2.79x10^-4^ | 93% (13/14) |
| F | 0.08 | 14% (1/7) |
| HN | 0.57 | 40% (4/10) |
| L | 0.08 | 55% (12/22) |
| non-coding regions | NA | 93% (13/14) |

**Supplementary Table 7**

| NP | | | M | | | HN | | | L | | |
| --- | --- | --- | --- | --- | --- | --- | --- | --- | --- | --- | --- |
| Codon change | **Amino acid change** | **%** | **Codon change** | **Amino acid change** | **%** | **Codon change** | **Amino acid change** | **%** | **Codon change** | **Amino acid change** | **%** |
| c.1318G>A | p.Asp440Asn | 10.1 | c.10T>C | p.Ser4Pro | 6.7 | c.331T>C | p.Leu111Leu | 10.9 | c.6179T>C | p.Leu2060Pro | 6.2 |
| c.1445T>C | p.Phe482Ser | 6.6 |  |  |  | c.332T>C | p.Leu111Ser | 13.5 | c.6431T>C | p.Leu2144Pro | 6.1 |
| c.1502A>C | p.Asn501Thr | 5.8 |  |  |  |  |  |  | c.6548T>C | p.Leu2183Pro | 10.3 |
| c.1586T>C | p.Val529Ala | 43.9 |  |  |  |  |  |  | c.6571T>C | p.Tyr2191His | 6.7 |
|  |  |  |  |  |  |  |  |  | c.6575T>C | p.Leu2192Pro | 6.3 |
|  |  |  |  |  |  |  |  |  | c.6586T>C | p.Leu2196Leu | 10.1 |
|  |  |  |  |  |  |  |  |  | c.6720A>G | p.Glu2240Glu | 9.3 |
|  |  |  |  |  |  |  |  |  | c.6726T>C | p.Ser2242Ser | 7.2 |
|  |  |  |  |  |  |  |  |  | c.6737A>G | p.Asp2246Gly | 5.5 |
|  |  |  |  |  |  |  |  |  | c.6753A>G | p.Ile2251Met | 22.4 |

**Supplementary Table 8**

| Protein | Mutation | Predicted binding affinity (IC50) value  for wild type | Predicted binding affinity (IC50) value  for mutated type |
| --- | --- | --- | --- |
| Donor alleles | | | |
| M protein* | p.Tyr170His | 91 | 240 |
| M protein* | p.Val172Ala | 91 | 240 |
| HN protein* | p.Ser10Leu | 55 | 81 |
| L protein* | p.Asp657Val | 163 | 125 |
| L protein | p.Pro1845Ser | 655 | 9891 |
| Random alleles | | | |
| M protein | p.Leu37Pro | 3179 | 2977 |
| M protein | p.Tyr140His | 1297 | 1598 |
| M protein* | p.Leu193Pro | 95 | 33 |
| HN protein* | p.Pro488Leu | 73 | 38 |
| L protein | p.Asp657Val | 1177 | 832 |
| L protein | p.Gln1867Glu | 1515 | 2837 |

## Supplementary Figures

**Supplementary Figure 1**

Read coverage (blue line) depth of the brain biopsy deep sequencing data mapped to the assembled sequence and is plotted across a genome schematic. (99.94% genome coverage, median coverage depth: 290, mean: 686, range: 1-5014). In green the read coverage depth of the NIBSC vaccine deep sequencing data mapped to a publicly available MuV^JL5^ reference (identifier: FJ211585) and is plotted across a genome schematic (100% genome coverage, median coverage depth: 706, mean: 818, range: 1-2369).

We observe a non-uniform coverage of the genome from the sequencing data, more specifically a decreasing gradient in the abundance of the transcripts produced for genes located further from the 3’ genome promoter. This is due to the fact that the RNA-dependent RNA polymerase (RdRp) recognises a single promoter at the 3’ end of the genome and can therefore only initiate there [1].

The difference in genome coverage between the brain and the vaccine virus may reflect the presence of mumps virus defective interfering particles[2].

**Supplementary Figure 2**

Full genome maximum likelihood phylogenetic tree of the viral sequence identified in the brain of the patient (in dark blue font) to all full mumps virus genomes found in GenBank (64 unique sequences, accession date 20 June 2015). The Jeryl Lynn strains are denoted in light blue color. The Bat Paramyxovirus was used as root.

**Supplementary Figure 3**

MuV^JL5^ strain (FJ211585) genomic structure and the single nucleotide polymorphisms observed in the brain virus genome. Missense polymorphisms are in red, synonymous are in green, non-coding in grey. The variants pre-existing in the vaccine sequence as minority variants are denoted with similar color coding but with dashed lines.

**Supplementary Figure 4**

Detection of mumps N protein in the CNS by immunohistochemistry. A & B. Low power photomicrographs showing abundant virus expression in neurons and neuropil in the grey matter. C. High power image of focal region from A demonstrating the presence of virus in neuron cell bodies and neuronal processes. D. Occasional virus positive cells were also detected in meningeal tissue on the surface of the brain.

**Supplementary Figure 5:** Minority variants in the MuV^JL5-^London genome. The amino acid changes are plotted along the genome, color-coded for each protein. The magenta-colored changes are occurring in non-coding regions of the MuV genome. Information on whether the changes occurred in predicted CTL epitopes is also provided.

**Supplementary Figure 6**: Visualisation of the multiple sequence alignment for 52 M proteins found in GenBank and the translated sequence of the M gene from the brain virus. Tyr140His is noted with a red arrow, while the 8 fixed *de novo* substitutions are noted with a purple arrow. In each page 50 amino acids are visualised.

**Figure 1**


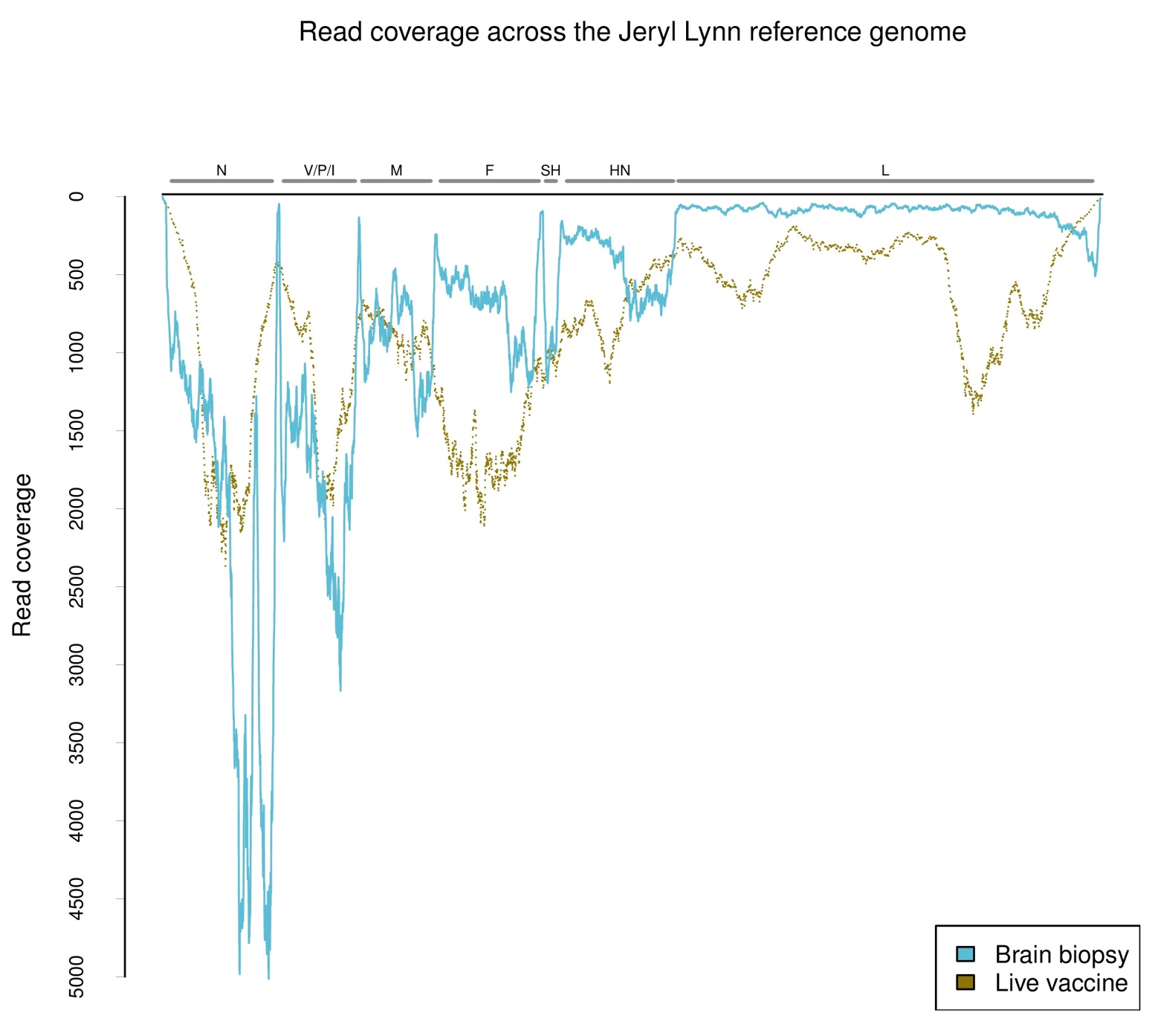


**Figure 2**


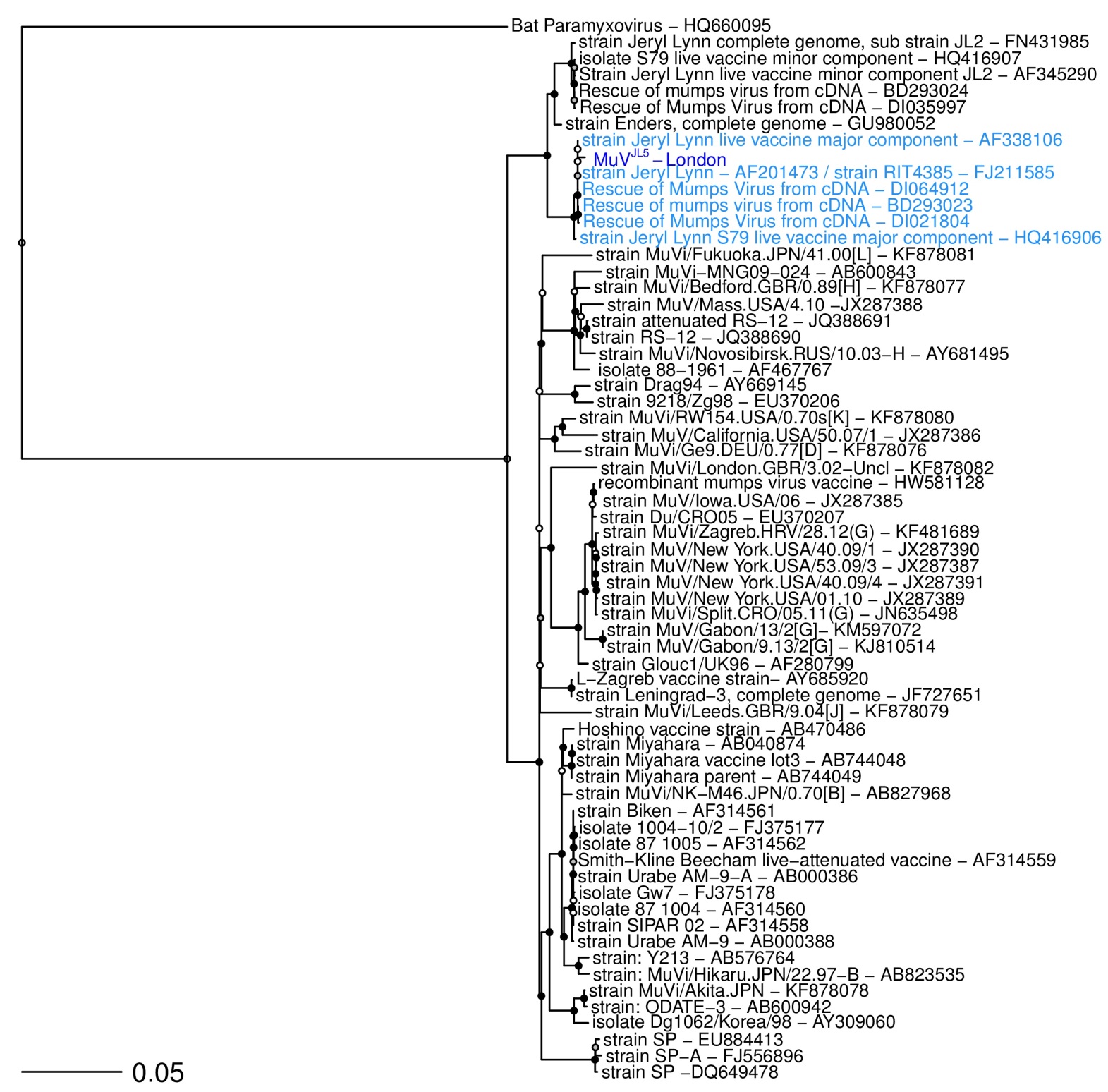


**Figure 3**

**
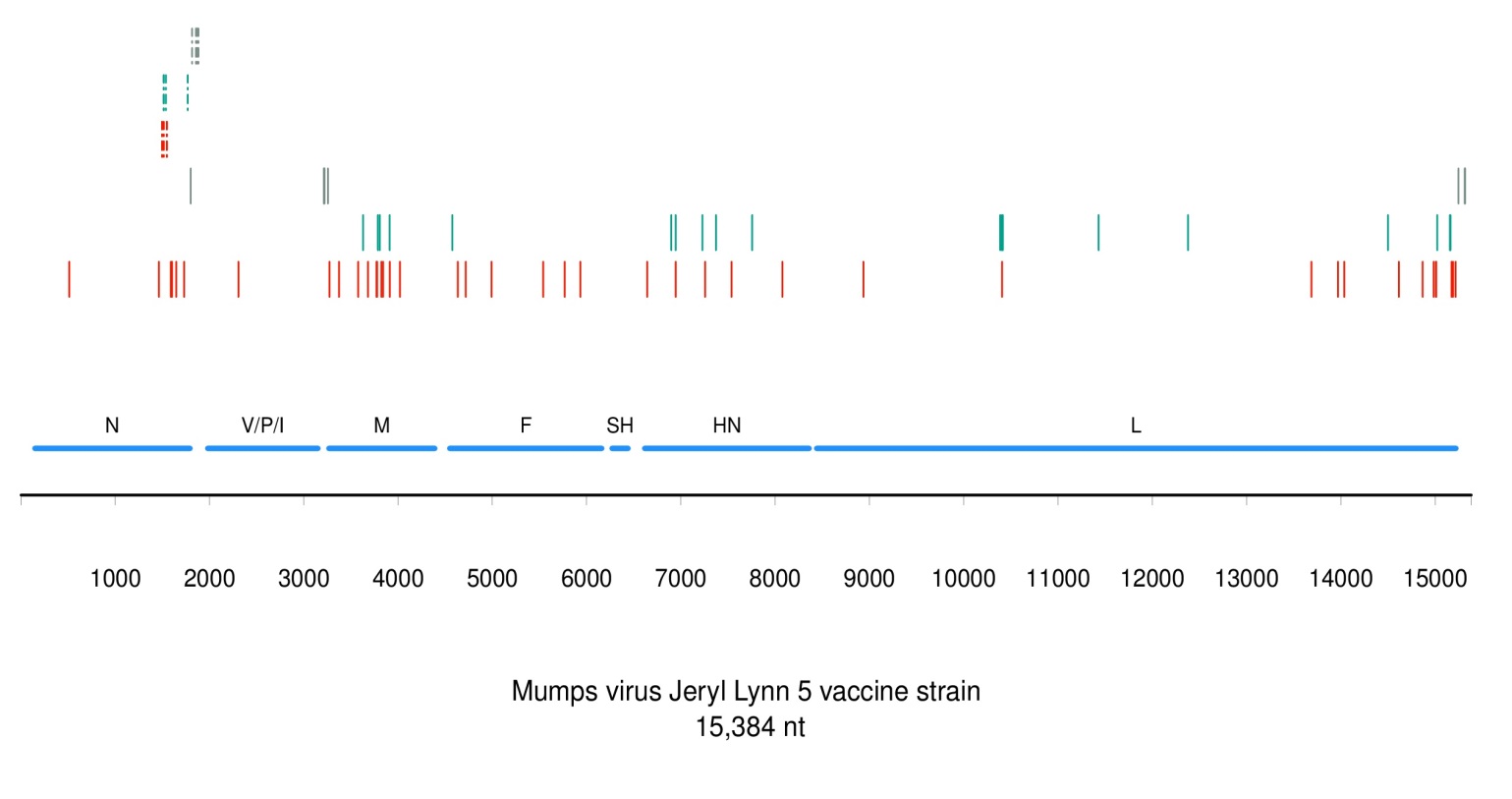
**

**
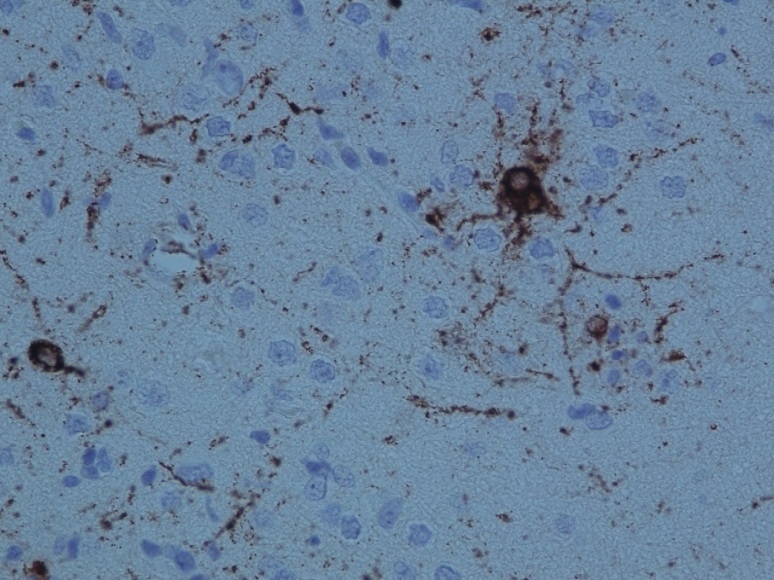

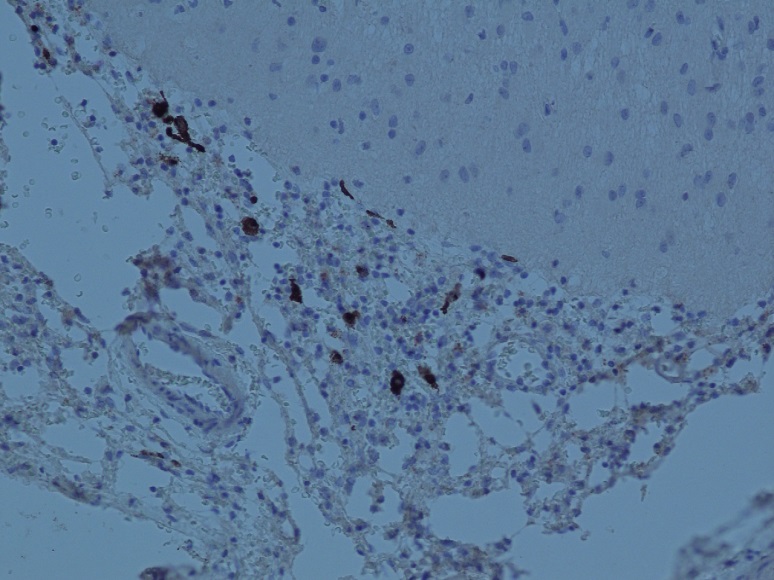

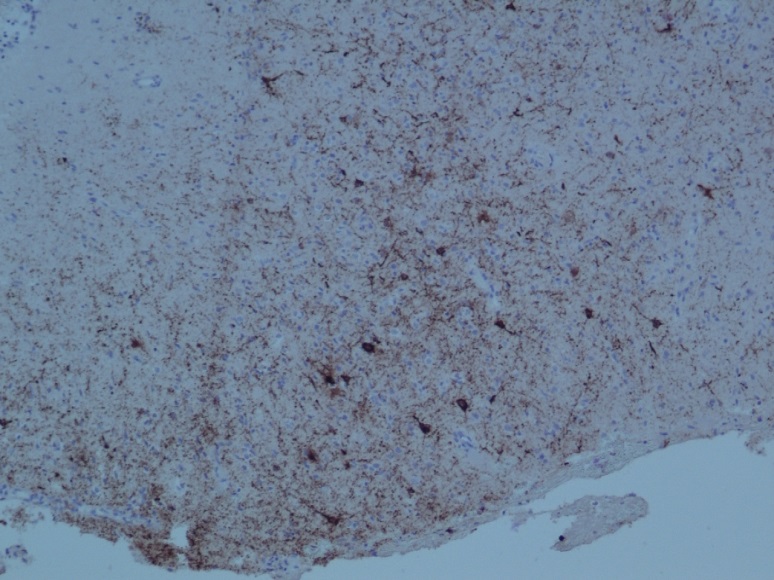

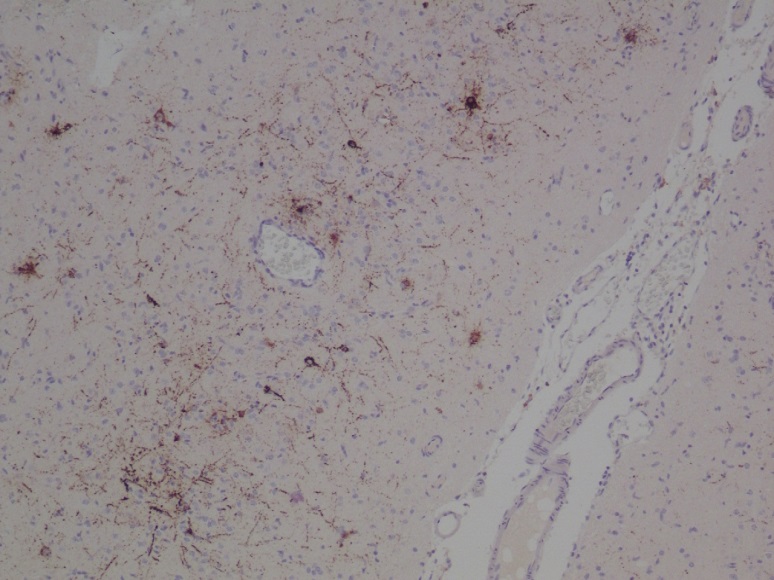
Figure 4**

D

C

B

A

**Figure 5**

**
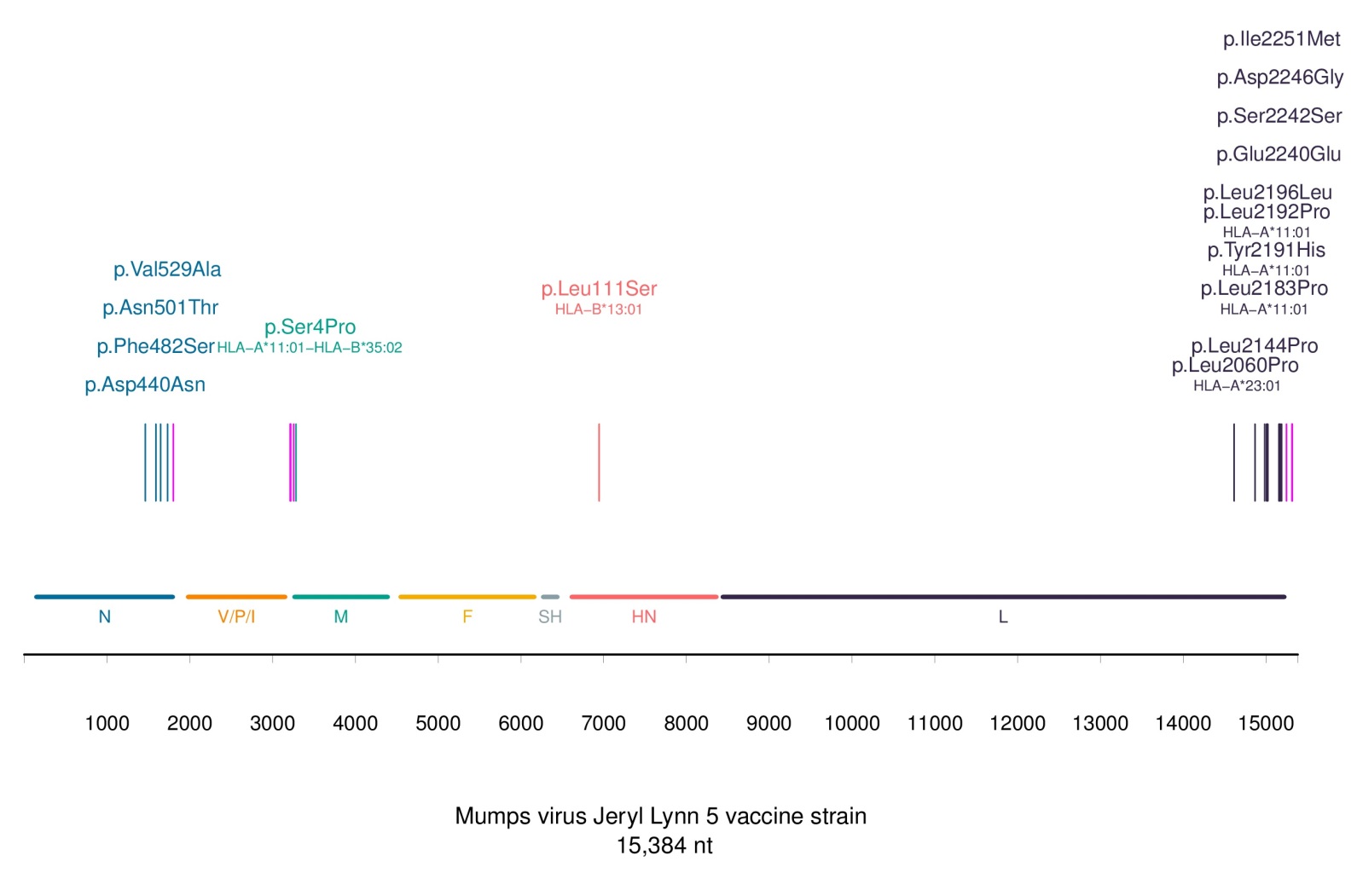
**

**Figure 6**
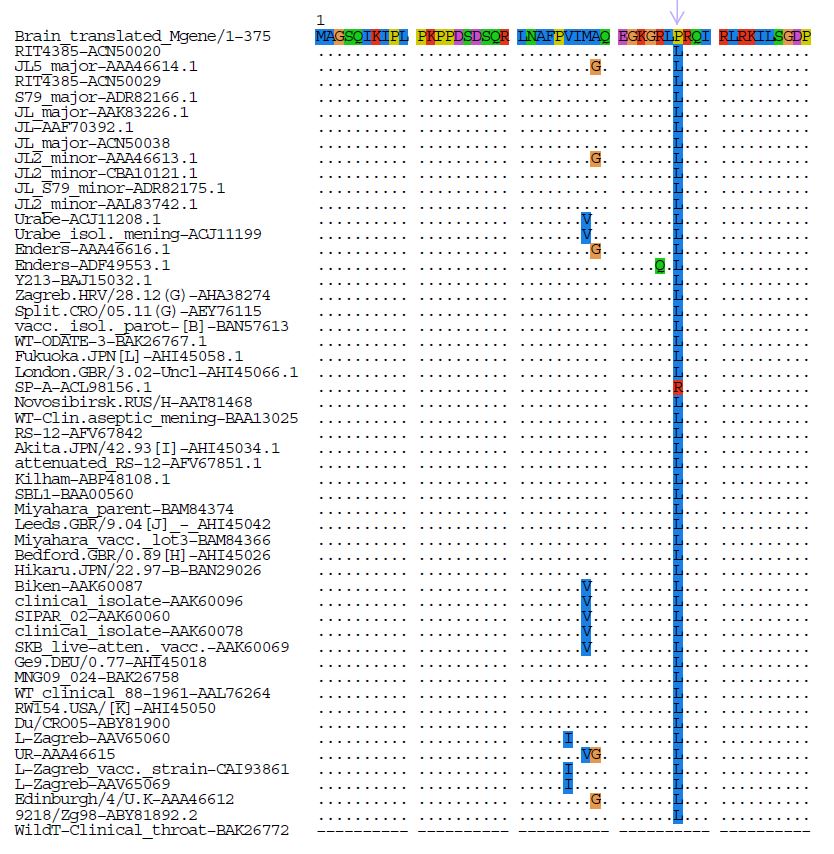


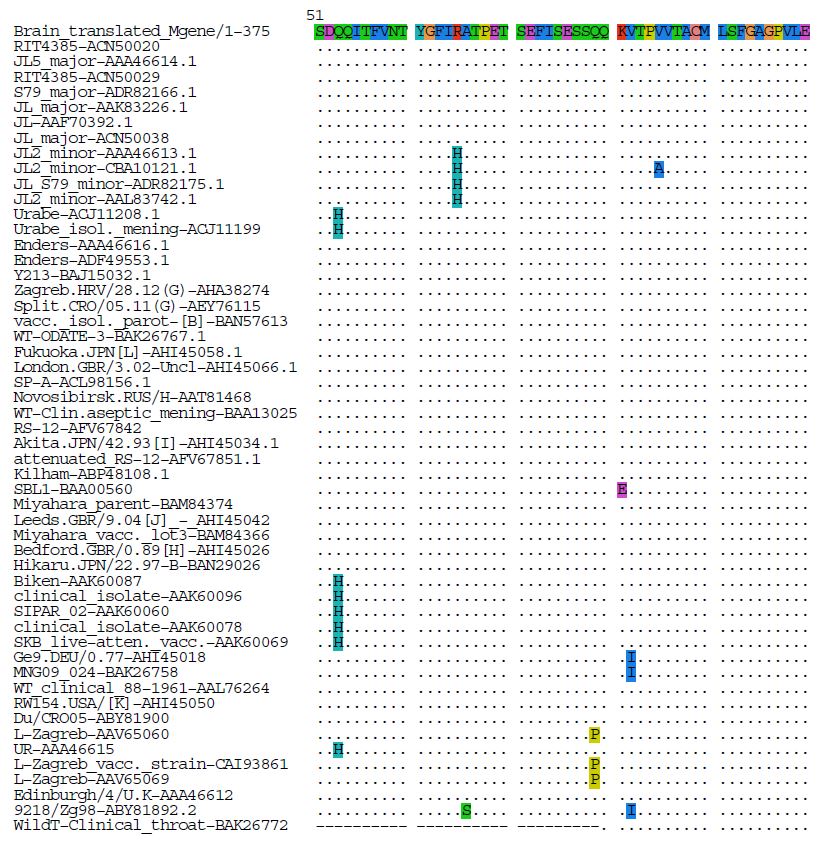


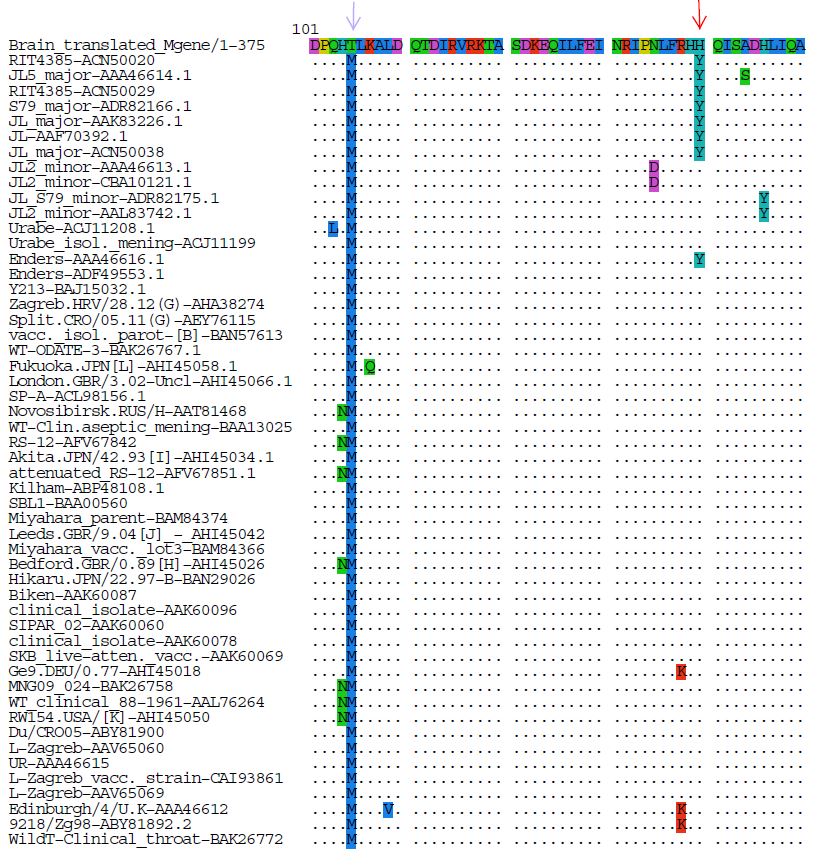

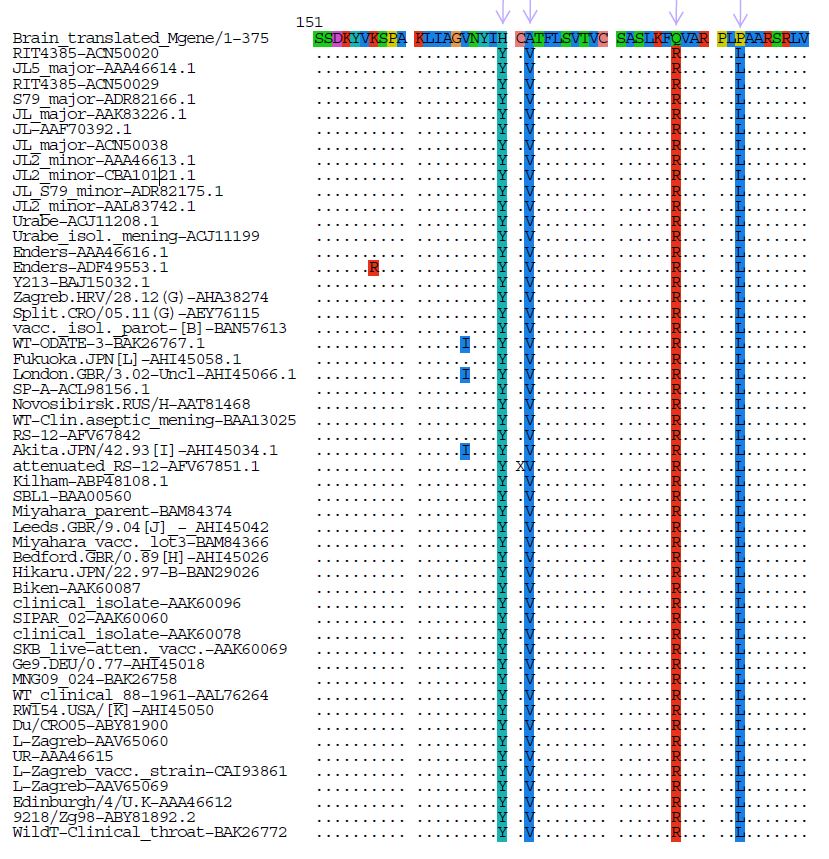

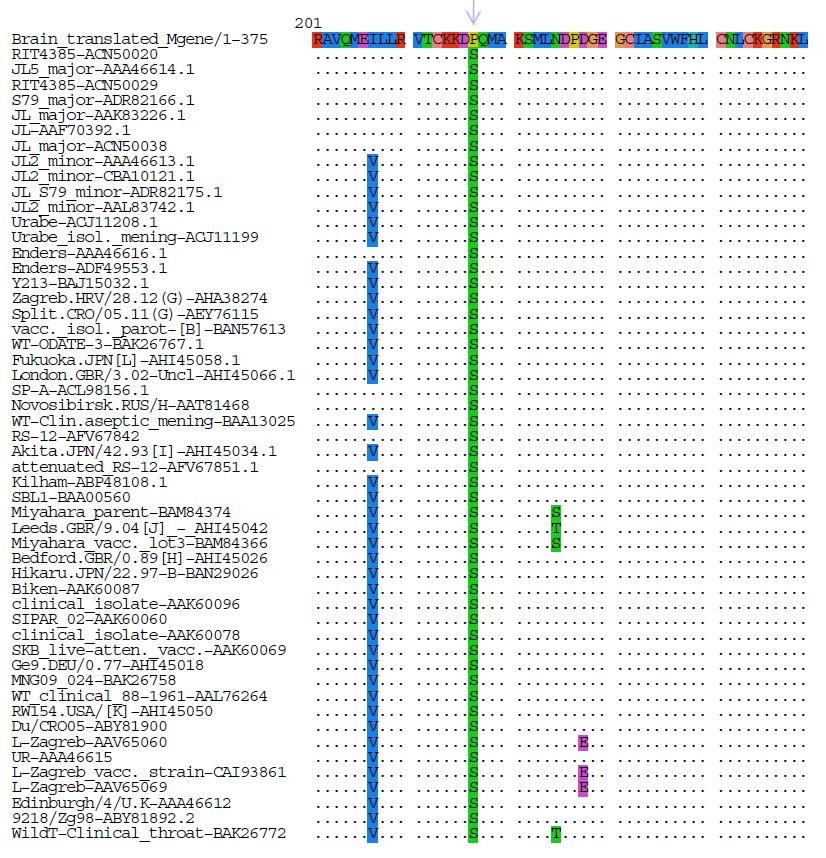

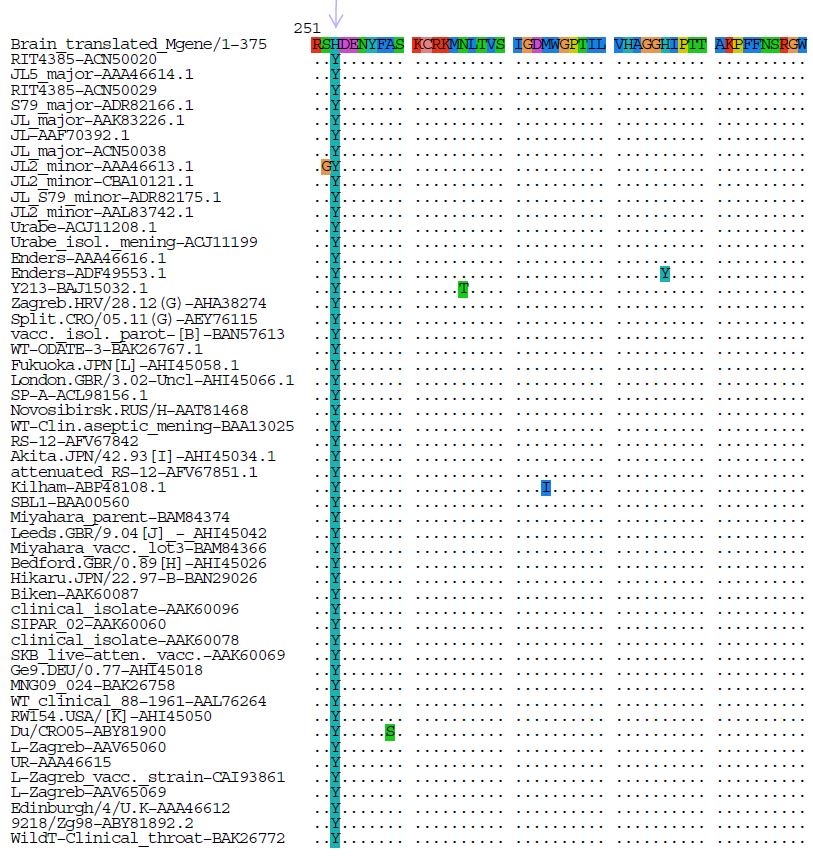

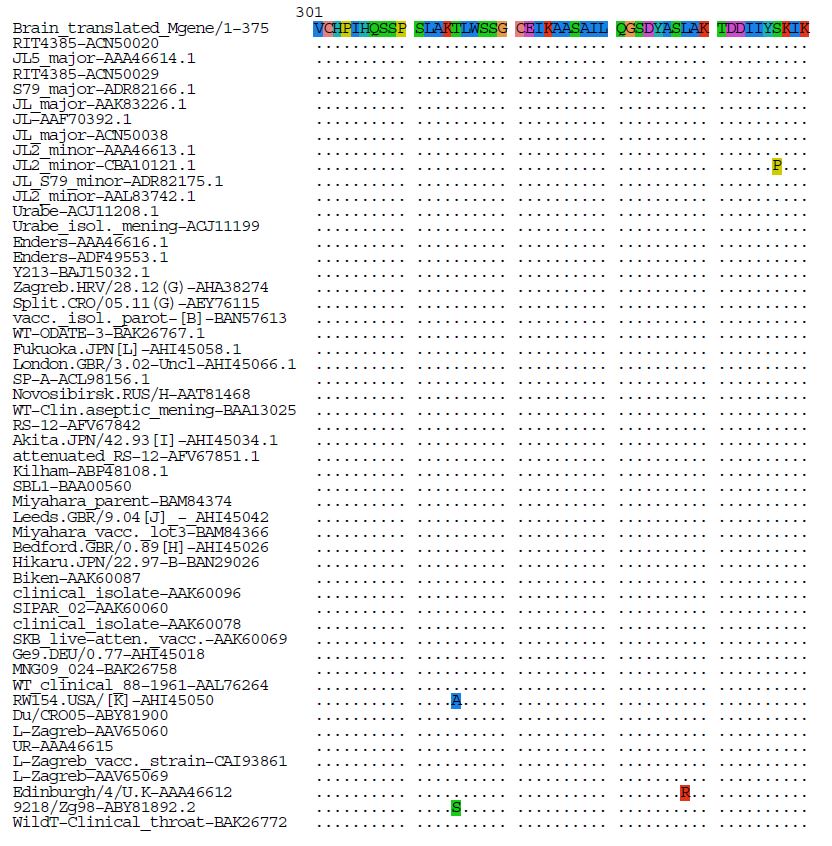

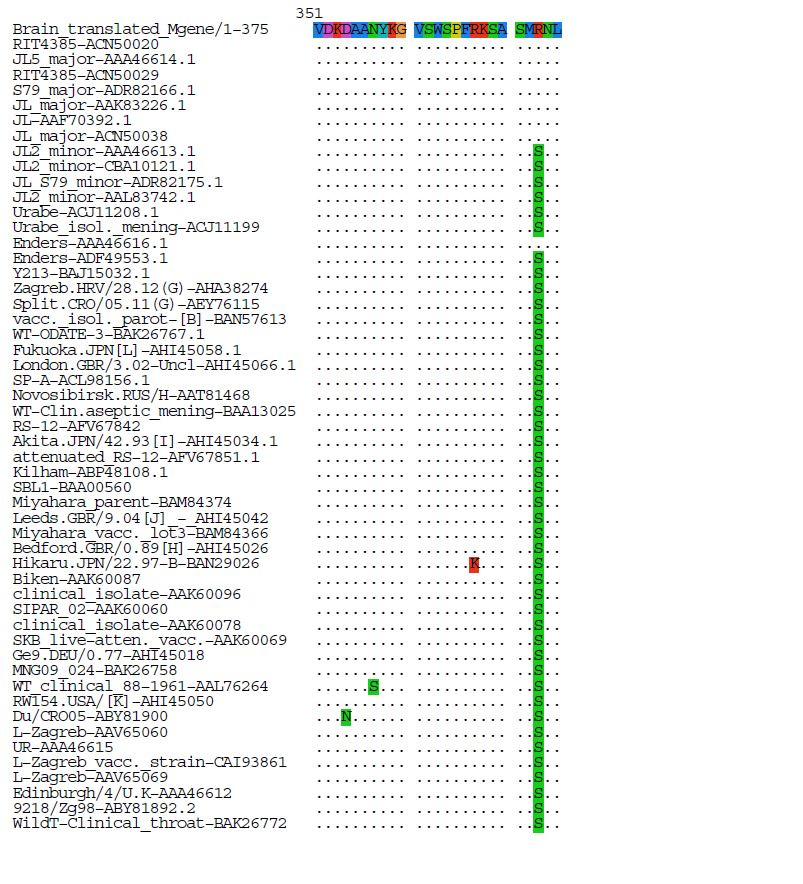


1. Rubin S, Vandermeulen C (2011) Mumps. In: Samal SK (ed) Biol. Paramyxoviruses. Caister Academic Press, pp 5–36

2. Šantak M, Markušić M, Balija ML, Kopač SK, Jug R, Örvell C, Tomac J, Forčić D (2015) Accumulation of defective interfering viral particles in only a few passages in Vero cells attenuates mumps virus neurovirulence. Microbes Infect 17:228–236. doi: http://dx.doi.org/10.1016/j.micinf.2014.11.006
